# Spiritual Counseling During the COVID-19 Pandemic in Italy: a Qualitative Study

**DOI:** 10.1007/s11089-022-00996-3

**Published:** 2022-02-03

**Authors:** Barbara Marchica, Francesco Rosellini, Erika Iacona, Michael Alexander Wieser, Ines Testoni

**Affiliations:** 1 Milan Insight School (MIS) Department of Spiritual and Pastoral Theology, Higher Institute of Religious Sciences of Milan (ISSRMI), Via Cavalieri del Sacro Sepolcro 3, 20122 Milan, Italy; 2 Department of Philosophy, Sociology, Education and Applied Psychology (FISPPA), University of Padova, Via Venezia 14, 35131 Padova, Italy; 3 Department of Psychology, Alpen-Adria-Universität Klagenfurt, Universitätsstrasse, 65–67, 9020 Klagenfurt am Wörthersee, Austria; 4 Emili Sagol Creative Arts Therapies Research Center, Faculty of Social Welfare and Health Sciences, University of Haifa, Haifa, Israel

**Keywords:** COVID-19 pandemic, Spirituality, Religiosity, Spiritual counseling

## Abstract

The COVID-19 pandemic has created profound upheavals in today’s society, accompanied by psychological effects. The discomfort experienced during the pandemic accompanied by the increased availability of time has offered many people the chance to reconnect with their spiritual dimension, which is considered a vital resource in managing the stress produced by the perception of risk to their health. This study addresses the motivations that led research participants to choose to receive spiritual support via a 10-week training. The work also explores the changes perceived by the participants as they overcame the difficulties resulting from the pandemic. The research involved nine people between the ages of 19 and 59 who took part in an online experience focused on the spiritual dimension. Almost all the participants came from an area in Northern Italy most affected by the pandemic. A qualitative research design was used, with semistructured interviews designed to understand participants’ views on the topic under investigation. The areas that emerged from the interviews concerned the motivations that led the participants to choose a spiritual support process, the role of spirituality in daily life, and the changes participants experienced after the conclusion of the experience related to managing the stress caused by the pandemic. In agreement with the existing literature, the results show that spiritual support can be useful in counteracting the negative effects of the pandemic, producing improvements in the quality of life.

There has been increasing interest in the literature on spirituality and religiosity and how these two dimensions positively affect people’s quality of life (Pargament et al., 2013; Testoni et al., 2019a, b). Spirituality and religion do not necessarily share the same psychological traits. Spirituality is about an individual’s ability to access the dimension of transcendence. In contrast, religion is considered the space in which spirituality is entrusted to language and ritual regulation (Pargament et al., 2013). However, spirituality and religiosity strongly influence people’s beliefs, attitudes, emotions, and behavior (Roman et al., 2020). Because many religions profess a healthy lifestyle, encourage social interactions, and offer a positive view of the future after death, believers and practitioners adopt less unhealthy habits and enjoy better social support, which limits the dangerous effects of stress (Helm et al., 2000; Levin & Chatters, 1998). In a study conducted in Kuwait and the United States, Abdel-Khalek and Lester (2010) showed that, in both cultures, religiosity correlates positively with several measures of subjective well-being and negatively with depressive symptoms.

Terror management theory, a psychological-social theory proposed by Pyszczynski et al. (2015), exposes how religious and cultural defenses against death anxiety influence altruistic and moral behavior (Jonas & Fischer, 2006; Testoni et al., 2018). They also serve as a form of defense against anxiety as they allow humans to answer existential questions, thus giving them the perception of having a certain level of control over their destiny and reducing their fear of death (Testoni et al., 2017a, b). Religion and spiritual life appear to be closely related to health, particularly concerning quality of life and resignation toward illness, both during a prolonged terminal phase and for shorter periods of convalescence (Jim et al., 2015).

In academia, there has been debate about the value of religious and spiritual competencies in the psychological domain and the importance of the interplay between these two domains in promoting people’s well-being. According to Harris et al. (2016), who summarized studies on the desires and expectations of people who rely on mental health experts, most of the literature agrees that those entering the world of psychotherapy often bring spiritual or religious issues into the consultation.

This is where pastoral counseling (or spiritual counseling) comes in. As its goal is the convergence of psychology and theology, pastoral counseling stands as a unique therapy model (Pedhu, 2020). Therefore, spiritual counseling is a practice aimed at improving quality of life in relation to socio-contextual, family, bereavement, and medical situations. Pastoral counseling addresses the needs of people who engage with mental health professionals while being able to welcome and reflect on their faith, simultaneously promoting psychological and spiritual well-being in a sort of “dual language” (Maynard & Snodgrass, 2015).

For Saguil and Phelps (2012), assessing and integrating a patient’s spirituality in the health care encounter can build trust and rapport, expand the doctor-patient relationship, and increase effectiveness. Practical outcomes may include improved adherence to physician-recommended lifestyle changes or treatment recommendations. Plus, addressing spiritual issues may allow patients to tap into an effective source of healing or adaptation.

Roman et al. (2020) sought to understand whether and how spiritual care might function as a coping strategy for practitioners and families during the global COVID-19 pandemic. The COVID-19 pandemic has intensified feelings of loneliness and social isolation due to lockdowns, the need for distancing, and the limits on social relationships to prevent the spread of infection (Hartt, 2020; Pierce et al., 2020).

In addition to all of these dramatic factors, other dimensions emerged that compounded the suffering of the bereaved, including the inability to accompany loved ones at the end of life and the inability to perform funeral rites (Cardoso et al., 2020; Testoni et al., 2020). These limitations largely prevented the possibility of using religion as a tool for processing suffering.

Studies have confirmed that healthcare professionals who provide spiritual care contribute significantly to improving the overall well-being of their patients (Melhem et al., 2016; Peltzer, 2009). Spiritual care is considered a life-enhancing factor and a resource for coping with adversity, enabling patients to cope with the most challenging and stressful times more effectively and increasing their hope for the future (McSherry et al., 2004).

Research has shown that the role that spiritual care providers assume plays a vital function, especially when families face challenging health risks and the prospect of palliative care (Wall et al., 2007). A study by del Castillo (2020), consistent with insights from recent studies on health, spirituality, and COVID-19, reiterates the need for holistic care; this means including, among other things, spiritual care, which research shows positively affects people’s well-being.

## Aims

The present research aimed to investigate what motivated the participants to choose a path of spiritual support, taking into consideration the importance that religion and spirituality assumed in the lives of the respondents. We wanted to understand participants’ expectations concerning the path undertaken and the changes perceived, that is, whether they considered support to the spiritual dimension valuable and helpful in overcoming the relational difficulties and social distancing due to the restrictions imposed by the outbreak of the COVID-19 pandemic.

## Participants and methodology

This study used qualitative research and a thematic analysis (Testoni et al., 2020). The study was inspired by the grounded theory approach (Corbin & Strauss, 1990; Glaser & Strauss, 1967). Grounded theory is a research approach that originated in the social sciences, and it intends to generate a theory based on the data and shaped by the opinions of the participants, thus moving beyond description and toward a theoretical explanation of a process or phenomenon without testing an existing hypothesis (Turner & Astin, 2021). Our research design adopted the narrative perspective, cited in the literature as the most reliable methodology for investigating people’s biographies (Testoni et al., 2020).

Semistructured interviews were used for data collection and were specifically created to explore participants’ views on the topic of interest. Semistructured interviews consist of closed and open-ended questions, often accompanied by follow-up questions focusing on the why or how of a particular respondent’s answer. Dialogue can build around topics that emerge naturally, rather than slavishly adhering to literal questions like a standardized survey, and delve into totally unforeseen issues (Adams, 2015).

The process of conducting the interviews consisted of two phases, one month apart and covering different topics. The first interview was to investigate what had motivated the participants to seek out such a path, the expectations they had from this support experience, and the role religion and spirituality played in their lives; the second was about the changes perceived by the interviewees and how this path had been effective for them, including in the aftermath of the COVID-19 pandemic. The interviews were conducted online on the Zoom platform and lasted an average of 45 min. All interviews were recorded to allow for later verbatim transcription.

The next phase involved the data analysis, performed using thematic qualitative analysis; this allowed the sources to be examined for main concepts or themes. The work then focused on an in-depth reading of the correspondence between the texts and the categories to identify central themes and any other subthemes (Testoni et al., 2016). The process allowed for the detection of both predefined categories and categories that emerged as unexpected topics, which only became clear as the analysis proceeded. The last step was further divided into parts: coding the data, verifying emerging understandings, searching for alternative explanations, and writing the report (Testoni et al., 2019a, b).

The study followed the American Psychological Association’s Ethical Principles of Psychologists and Code of Conduct and the principles of the Declaration of Helsinki. It received research ethics approval from the Ethical Committee for the Psychological Research of the University of Padua (reference: 1D0278C216A4AF1B831782B58E31F3B6). Participants provided written informed consent before participating in the study.

The participants who decided to participate in the study were selected from among people who were accepted the opportunity to participate in a spiritual counseling course led by Professor XXX (Identity and Purpose of Pastoral Counseling), whose method integrates Bernard Lonergan’s epistemological theory and the practice of spiritual counseling (Speed Method_MIS®). The Speed Method offers concrete and practical help to those who want to achieve their goals and personal, family, and professional serenity. The training (ten meetings) enhances the level of awareness and consolidates interpersonal skills (life skills) using the four steps of the Speed Method (feeling, thinking, discerning, and choosing; Marchica, 2019). The Speed Method offers four fundamental benefits: effectiveness of results, timeliness of learning, self-training of the cognitive process, and awareness of one’s own spiritual intelligence (Marchica, 2018).

The group of participants who took part in the research consisted of nine people between the ages of 19 and 59 (M = 41.89, SD = 16.72). All participants followed the same path of support for the spiritual dimension, led by the same professional. Of these, seven were female and two were male. Only two of the participants were married, and two had children. Eight described themselves as believers, and one described themself as a nonbeliever. Six of the believers actively participated in religious practices.

All the objectives of the research were explained to the participants in detail, as well as the methodology of the analysis. They were asked for permission to record the conversations, transcribe their responses, and analyze their content to study the phenomenon. We guaranteed anonymity regarding the content of the texts obtained, and only those who gave written and signed consent participated in the research. All the names mentioned below are fictional (pseudonyms), and the quotes have been slightly modified to prevent any possibility of identifying the participants.

## Results

The thematic analysis allowed several macro areas to emerge within the interviews related to the motivations that had prompted the participants to seek spiritual support; the importance that religion and spirituality had in their lives; the changes that the interviewees perceived as a result of the spiritual support training they participated in; and the role that their spiritual path has had in helping them withstand the distance and isolation caused by COVID-19.

## The first area of significance: motivation

The motivations reported by the participants related to their decision to seek spiritual support were mainly related to a desire to increase their self-esteem and improve their relationship management.

### Self-esteem

The first aspect, the desire to cultivate one’s self-esteem, seemed to be the most common motivation within our group of participants. Vincenzo, a 56-year-old man who worked as a parish priest, testified: “I am working, and I will have to work... on my self-esteem, certainly this..., these are the elements on which, in perspective, I think are most important ones to work on here.”

Renzo, a 31-year-old man who worked as a religion teacher, agreed with what Vincenzo said: “I have more serenity in facing these problems. At the beginning I had lost it a bit, and moreover my self-esteem had dropped, therefore Barbara [Machica] and I worked on this sense.”

Margherita, a 22-year-old girl who worked as a bartender and had a passion for photography and art, seemed to agree when she said that in the past she’d had some experiences that had impacted her low self-esteem. Now, she turned to this spiritual support with hope: “Then I believe, and hope, that I can work especially on, as I said before, on boosting self-esteem so that I stop comparing myself to others.”

Renata, a 52-year-old woman who worked as an accountant, in line with the participants reported on so far, told us how, following a particularly stressful period: “I was saying: but I can’t be like this, I have to do something, and I don’t know what, what parts of me to work on... on giving myself value, on my low self-esteem.”

### Relationship management

Another aspect of the motivations that pushed our participants to choose a path that supported their spiritual dimension that emerged with significant frequency was the desire to improve the management of relationships that they considered dysfunctional or problematic.

Rita, a 58-year-old woman who was a charity nun, explained that she had a constant thought that she could not ignore: “I was at a bit of a standstill where I was wondering:... I know all the things, but how do you make that leap to gain those precise skills in daily life so that I avoid stumbling? In some difficult relationships, in some things I couldn’t understand, I was missing that very how.”

Vincenzo said how important it was for him to be able to understand how to best manage his relationships in order to improve them:

The first is the question still unanswered on, so to speak, on the clarification of the functioning of my relationships, not of the general relationships in theory that this is quite easy to find, and then the improvement of existing relationships, those that I currently live, . . . to be able to, once I understood the functioning, improve the relationships; I had not found an answer in the paths that I had done previously.

Alessandra, a 19-year-old woman who worked in a marketing company, reported about her behaviors: “Let’s say that they were nonfunctional both to me as a person, in the sense that they worsened the course of my day, and others: with friends, my boyfriend and with colleagues as well the environment I was in contact with, it’s like when you’re angry... others perceive this thing.”

Morena also explained that she felt the need to deepen some techniques to improve her relationships: “Initially,... it seemed to me that at that moment I needed to sharpen some listening and communication techniques... with the people I come in contact with.”

## The second area of significance: importance of religion and spirituality

In this area of significance, the importance that religion and spirituality have in the participants’ lives emerged.

When Morena was asked how her relationship with religion and spirituality began and how important these aspects were in her life, she said,

I come from a Catholic family, so until a certain age it was taken for granted to do certain things like go to Mass, let’s say take on certain practices, then from a certain point I moved away completely . . . and then when I was 19 years old I got closer . . . through a missionary, let’s say, a missionary who was ordained right there where I lived, and his figure, his passion and all that he represented, disrupted my life, and from there a slow but conscious rediscovery of what it meant to be a believer for me started. . . . And from there a crescendo until I became a missionary, and so here we touch the ‘fulcrum’ of my life: I can’t live my life without this relationship with the absolute.

This path of spiritual growth, then, can also be found in Letizia’s story as a 27-year-old woman who worked in an advertising agency:

When I was eighteen or nineteen, I really felt a call, let’s say, a need. It was a bit of a dark moment, and I felt like going to church, trivially. It was December 25, Christmas, and from there, I began to create a more concrete relationship with God. . . . Now I trust myself much more, then I went to Medjugorje [an unofficial place of Catholic pilgrimage located in Bosnia and Herzegovina] two years ago, and that changed my vision.

Stefania, a 53-year-old woman who worked as an art history teacher, seemed to agree, and she told how, through her life experiences, her faith and her spirituality had developed:

I grew up in this family that accompanied me on my spiritual path, then I found my dimension over time, thanks to my experiences. The first one was surely a traumatic one, the loss of my father, so I already had an inclination towards spirituality, faith, and that certainly strengthened it. . . . A second experience was that of being a parent, and therefore of being aware that your spiritual and pastoral experience can continue through your child. . . . Another experience that further strengthened this dimension of mine was certainly the cancerous illness that I had . . . and that further enhanced this belief of mine.

## The third area of significance: changes

Two aspects of change were distinctive for the frequency with which the participants reported them. The two changes that were perceived most by respondents were increased awareness and improvements in relationship management.

### Awareness

Several participants reported how an increase in their level of mindfulness was one of the most noticeable changes as they were on their spiritual support journey. For example, Vincenzo recounted how he perceived his change: “In the acquisitions that I received, the question of time, of duration, therefore of the possibility of activating in daily life the awareness that I have acquired,... made me realize that now I have found a sort of stability, of balance.”

Similarly, Stefania also spoke of how her level of awareness had changed: “Especially at the beginning, I noticed... a greater awareness of this identity that is ours and that is very complex, and therefore a part that I had recognized but was not always made visible in the awareness... this way of approaching has become a bit more concrete.”

Renzo also testified that his main change was in the acquisition of greater awareness: “I internalized some things that she [the professor] often repeated. I remember her saying that sometimes you just need to expand the field of consciousness... which, said in other words, means looking at the problem with different eyes or having greater awareness of the events or the problems; this was the primary thing: I can say so.”

### Relationship management

Regarding the improvements that our participants reported in their testimonies, in terms of relationship management Rita recounted: “Clearly, I see some results within the relationships, and this is what I was looking for in short, among the approaches... I can now manage even the most complex relationships, with the most intrusive people... relationships in which I used to get caught up, and I didn’t know how to get out, now it’s a whole different story.”

In the same way, Margherita explained that now she can enjoy relationships and moments that previously she was not able to value: “Basically, also regarding the outings among friends, that before I thought are just usual outing among friends, I shoot some crap and [go] away. Instead, now I am able to enjoy the moment much more.”

Vincenzo also wanted to reveal how the changes that had come from this spiritual support training had made him more stable and determined, even in everyday relationships: “I realize that I am more self-centered and less (less is a big word) but certainly less fickle in relation to what happens around me... in the relationships I deal with every day.”

## The fourth area of significance: the help spiritual counseling provides in coping with COVID-19

Spiritual support seems to have played an essential role from the point of view of the support it provided to the interviewees to bear the social distancing and the lack of relationships imposed by decrees caused by the expansion of the COVID-19 epidemic.

As Margherita said, the spiritual support training allowed her to put internal resources in place to avoid being overwhelmed by feelings of boredom and sadness due to the impossibility of meeting with other people: “Actually, it helped me... to interface with this situation, in the sense of not depressing myself in bed or on the couch and to push me to do things like go to the park, take pictures, which is my passion. The drive to do something that was of interest to me overpowered everything else.”

Rita agreed and underlined how, in her experience, although she would have preferred to carry out the course in person, the spiritual counseling had been a real help: “Ah well... yes, yes it has helped me a lot.... It was a great help, I keep saying that it would have been better in person, but luckily it was available... it was a great support.”

Vincenzo agreed with what Margherita and Rita said, emphasizing that the lack of relationships was a very difficult aspect for him. Moreover, he also added the aspect of time management, which, before starting this training, had proven to be slightly difficult for him: “Yes, yes, I tell you absolutely yes. Dealing with two major labors and the lack of relationships for me was dramatic. Dramatic means that I really struggle to interact that I from in front of a screen, so this thing I missed so much. Certainly, having to center myself has broadened my mind. The other direction that, for me, is fundamental is time management. In the first part of the total lockdown, managing time, the 24 h, was really, it was very tiring for me, scattering, tiring, closed in the house.”

## Discussion

The findings from this research agree with the literature regarding the positive effects of spirituality and spiritual support in managing the most stressful life experiences. The study highlights that several needs lead people to seek spiritual support.

In this study, low self-esteem, and therefore the need to increase and cultivate self-esteem to feel better about themselves, along with the desire to manage certain relationships that were unsatisfactory and complex, played a significant role. This is a significant result because the existing literature has not valued this aspect; it should be taken into consideration. It is important to underline how terror management theory has highlighted the relationship between the death anxiety caused by mortality salience and the need to strengthen self-esteem. When mortality salience increases anxiety levels, people need to find strategies to increase their self-esteem.

Adherence to religious practices helps with this. This first result thus confirms what has been highlighted by empirical studies carried out in the field of social psychology (Dechesne et al., 2003; Lifshin et al., 2021; Pyszczynski et al., 2004). Arguably, the need to bolster self-esteem for some participants became cogent precisely during the COVID-19 pandemic.

The specific aspect that this research highlights is the possibility of offering people a new awareness of themselves and of their spirituality, thanks to the methodology used in the proposed path. Therefore, spirituality is not to be seen as an intimate level that is an end in itself but as an opportunity to create better relationships with oneself and others. In light of the testimonies collected, spiritual and religious resources become an opportunity to improve and contribute to building qualitative relationships in an edifying and decisive way.

Based on the literature and also on our study, spirituality and religiosity enable people to cope more effectively with the stresses related to daily life (Testoni et al., 2019a, b), even those related to concerns about their health status (derived, for example, from the fear of infection with COVID-19; Testoni et al., 2021) because they help to reduce the fear of death. In particular, religion fuels the belief in an afterlife such that death constitutes a passage to another dimension (Bianco et al., 2019).

Our results show that those who use spiritual counseling consider religion a central aspect of their lives The literature has already highlighted the importance of providing opportunities for deepening and solving religious problems, such as the study by Rose et al. (2001), according to which having significant spiritual experiences during one’s life makes people more likely to consider the merits of issues related to the religious sphere and to evaluate their implications.

Regarding perceived changes, our results show that spiritual support helps people become more mature and gain greater self-awareness and the ability to solve relational problems that help to give meaning to life and, as noted by some scholars (Testoni et al., 2017a, b; Yilmaz & Cengiz, 2019), that spiritual well-being plays a very important role in managing stress and anxiety about death in a meaningful way (Testoni et al., 2021).

Therefore, spiritual counseling is considered influential by those who use this service. This type of support meets the needs of those who feel the need to engage with someone who can also understand the meaning of their faith, responding to issues that others are unable to consider. This type of matching increases psychological well-being (Maynard & Snodgrass, 2015) and self-awareness (Pandya, 2020). All of this allowed the participants in the present study to reduce the anxiety produced by the COVID-19 pandemic by counteracting feelings of loneliness fear, and boredom. This corresponds with Fraenkel and Cho (2020), who pointed out that it seems necessary to access a higher dimension when faced with an unpredictable situation.

Indeed, the literature shows that it is crucial to reflect on higher values that can be accessed through spirituality and religion. According to Dreyfus and Wrathall (2014), these values can often be examined superficially, but sometimes extreme conditions force people to examine them more deeply to derive principles that can serve as a compass to guide them through unfamiliar terrain in life (Fraenkel & Cho, 2020).

Our study thus confirms that the spiritual dimension is potentially an essential resource for stemming the effects of stressful and traumatic events (González-Sanguino et al., 2020), which the COVID-19 pandemic certainly was.

## Conclusions

This study confirms the importance of spiritual counseling. Participants confirmed that the spiritual support they received played an important role in improving their quality of life by nourishing their propensity to strengthen their bonds with others even during the pandemic. In fact, it emerged that the spiritual support training helped participants resolve deep problems regarding the meaning of life and the trials it unexpectedly holds, transforming negatives into positives, including the pandemic experience.

The pandemic thus became an opportunity to get closer to the inner dimension and regain contact with a spirituality and a faith that some of the participants had set aside because of the frenetic rhythms of daily life but that, if tended to, could improve many aspects of their lives. Therefore, if lived in a conscious way, the spiritual and religious dimension can favor one’s relationship with oneself (the self-esteem process) and contribute to building qualitative relationships (life skills and relationship management).

## Limitations and future research

The main limitation of this study is the limited number of participants. A small corpus of interviews does not allow for inferences and, like all research that adheres to a qualitative research design, does not allow for the generalization of results. This study can be considered a starting point for further studies related to the outcomes of spiritual support. In future research, recruitment could aim for a more extensive and more diverse group of participants.
